# Essential oil of *Cyphostemma juttae* (Vitaceae): Chemical composition and antitumor mechanism in triple negative breast cancer cells

**DOI:** 10.1371/journal.pone.0214594

**Published:** 2019-03-28

**Authors:** Pietro Zito, Manuela Labbozzetta, Monica Notarbartolo, Maurizio Sajeva, Paola Poma

**Affiliations:** Department of Biological, Chemical and Pharmaceutical Science and Technology (STEBICEF), University of Palermo, Palermo, Italy; University Sains Malaysia, MALAYSIA

## Abstract

The genus *Cyphostemma* (Planch.) Alston (Vitaceae) includes about 150 species distributed in eastern and southern Africa and Madagascar. Some species are used in traditional medicine and their biological activities, including antiproliferative effects against cancer cell lines, have been demonstrated. To date no investigations on *Cyphostemma* essential oils have been carried out. Essential oils, which play important roles in plant defenses have been demonstrated to be active in the treatment of several human diseases and to enhance bioavability of other drugs. The aim of this paper was to identify the chemical composition of the essential oil of the leaves of *Cyphostemma juttae* (Dinter & Gilg) Desc. and to verify some biological activities on two triple negative breast cancer cell lines (MDA-MB-231, SUM 149), characterized by the over-expression of the transcription factor NF-κB. In the essential oil, obtained by hydrodistillation and analysed by gas chromatography-mass spectrometry, 39 compounds were detected and with phytol (30%) dominating the chemical composition. *C*. *juttae* essential oil reduced cell growth and showed a pro-oxidant activity in both cell lines. Moreover, *C*. *juttae* essential oil caused a substantial decrease of NF-κB activation and consequently a significant reduction of some NF-κB target genes. The present study shows for the first time the cytotoxic properties of *C*. *juttae* essential oil and highlight its availability to interfere with NF-κB pathway, suggesting a potential therapeutic use in triple negative breast cancers (TNBCs) of this essential oil.

## Introduction

The genus *Cyphostemma* (Planch.) Alston (Vitaceae) includes about 150 species distributed in eastern and southern Africa and Madagascar [1]. Some species from this genus are used in traditional medicine, e.g. roots of *C*. *junceum* to treat snake bites [2]. Interestingly, Ochawang’i *et al*. [3] described the use of *C*. *serpens*, alone or in association with other plants, in traditional medicine for the care of breast cancer. Udegbunam *et al*. [4] used root extracts of *C*. *vogeli* to investigate its anti-inflammatory effect on sore throat, cough and pneumonia in mice. The antiproliferative effects of water and methanolic extracts of four *Cyphostemma* spp. (*C*. *flaviflorums*, *C*. *lanigerum*, *C*. *natalitium*, and one unidentified species) on HepG2 cell line have been studied by Opoku *et al*. [5], but no chemical analyses of the matrices were performed. Cao *et al*. [6] found that *C*. *greveana* showed antiproliferative activities against the A2780 ovarian cancer cell line and identified one macrolide, lasiodiplodin, three sesquiterpenoids, and a new diterpenoid. Although from a chemical point of view *Cyphostemma* spp. have been investigated by solvent extractions, to the best of our knowledge no studies have been carried out on hydrodistillated essential oils and their possible biological activities.

The aim of this paper was to identify the chemical composition of the essential oil of the leaves of *Cyphostemma juttae* Dinter & Gilg (Desc.) obtained by hydrodistillation (HD) and analyzed by gas chromatography-mass spectrometry (GC-MS), and to verify some biological activities on two triple negative breast cancer cell lines (MDA-MB-231, SUM 149). Triple negative breast cancers (TNBCs) are highly aggressive, do not respond to conventional hormonal interventions due to the lack of the respective receptor targets, have chances of early recurrence, metastasize, tend to be more invasive in nature, develop drug resistance and they are also characterized by the over-expression of the transcription factor NF-κB [7]. The relevance of the NF-κB transcription factor in tumor biology is widely known, even in triple negative breast cancer.

## Material and methods

### Plant species

*Cyphostemma juttae* (Dinter & Gilg) Desc. (Vitaceae) is a tree-like succulent plant up to 2 meters tall that forms a massive caudex with thick branches. Stems and branches are covered in yellow and papery skin, peeling off with age. Leaves are deciduous, succulent, up to 20 cm long and 6 cm wide, and are produced in Summer during the vegetative season. This species grows in Namibia, it is adapted to very dry environments and it is also cultivated as ornamental in specialized collections.

### Plant material

Leaves of *Cyhostemma juttae* were collected in July 2017 from plants cultivated at the Botanical Garden of the University of Palermo. The plants were raised from seeds in 1984 and pot cultivated in the open with references code: Vitaceae N-146. The matrices were placed in paper bags and kept at -30°C for 24 hours before hydrodistillation. No specific permits were required for the described location and for the collection of plant material because the plants are part of the living collection of the Botanical Garden of the University of Palermo and the authors have access to that. The plant species used in the present study is not endangered and the IUCN category assessed is Least Concern [8]; the seeds were obtained and the plants raised before the Convention on Biological Diversity (CBD) entered into force on 29 December 1993 and therefore are pre-CBD specimens.

### Essential oil

Leaves (868 g) were hand-cut into small pieces (~ 2 cm) and hydrodistillated for 3 hours in a Clevenger-type apparatus, using *n*-pentane as collection solvent. The oil was dried by anhydrous sodium sulphate and stored at -30°C until chemical analysis and pharmacological tests. The essential oil yield was 17.36 mg (0.002%). To prepare the stock solution for biological studies 2 mg of essential oil were dissolved in 1 ml of dimethyl sulfoxide (DMSO).

### Gas chromatography-mass spectrometry

Essential oil sample was analyzed by GC-MS on a Shimadzu GC-MS-QP2010 Ultra equipped with an AOC-20i autoinjector (Shimadzu, Kyoto, Japan) and a ZB-5 fused silica column (5% phenyl polysiloxane; 30 m long, inner diam 0.32 mm, film thickness 0.25 μm, Phenomenex). One μl of diluted sample (5x10^-3^, in n-pentane) was injected at 280°C in a split ratio of 1:1, and the column flow (carrier gas: helium) was set at 3 mL min^-1^. The GC oven temperature was held for 1 min at 60°C, then increased by 10°C min^-1^ to 300°C and held for 5 min. The MS interface worked at 300°C, and the ion source at 200°C. Mass spectra were taken at 70 eV (in EI mode) from m/z 30 to 450. The GC-MS data were processed using the GCMSolution package, Version 4.11 (Shimadzu Corporation 1999–2013).

### Identification of compounds

Compounds were identified by using the mass spectral libraries [9], FFNSC 2, W9N11, and ESSENTIAL OILS (available in MassFinder 3) and the Kovats Retention Indices (KRI) of the compounds based on *n*-alkane series. We only considered compounds which had a mass spectral similarity more than 70% respected to those present in our digital libraries and which had a calculated Kovats index ± 10 compared to available data bases [9,10] and Nist11.

### Cell growth assays

Triple negative breast cancer cell lines (MDA-MB-231, SUM 149) were used. The human breast cancer cell lines MDA-MB-231 (ATCC: HTB-26—Rockville, MD, USA) and SUM149 (SUM149PT—Asterand Bioscience Detroit, MI) were kindly provided by Dr. Elda Tagliabue (Molecular Targeting Unit, Department of Experimental Oncology and Molecular Medicine, Fondazione Institute of Hospitalization and Scientific Care, National Cancer Institute, Milan, Italy) and were authenticated using the short tandem repeat profiling method in their Institute. The non-tumorigenic cell line 1-7HB2 (ECACC 10081201—Cancer Research Technology, London, UK) was kindly provided by Prof. Giulio Ghersi (STEBICEF Department, University of Palermo, Italy).

MDA-MB-231 cell line was cultured in RPMI-1640 and the SUM 149 cell line was cultured in DMEM/F-12 supplemented with insulin (5 μg/ml). 1-7HB2 cell line was cultured in DMEM low glucose supplemented with hydrocortisone (5 μg/ml) and insulin (10 μg/ml). All media were supplemented with 10% heat-inactivated fetal calf serum, 2 mM L-glutamine, 100 U/ml penicillin and 100 μg/ml streptomycin (all reagents were from EuroClone S.p.A., Milan, Italy; GE Healthcare Life Sciences, Logan, UT, USA). The cells were cultured in a humidified atmosphere at 37°C in 5% CO_2_. After obtaining the cells, the first passage carried out was assigned passage number 1. Cells with a narrow range of passage number (4 ± 6) were routinely tested for Mycoplasma contamination were used for all experiments.

The cells were seeded at 2 × 10^4^ cells/well onto 96-well plates and incubated overnight at 37°C. At time 0, the medium was replaced with fresh complete medium supplemented of essential oil at the indicated concentrations. Following 72 h of treatment, 16 μl of a commercial solution obtained from Promega Corporation (Madison, WI, USA) containing 3-(4,5-dimethylthiazol- 2-yl)-5-(3-carboxy methoxyphenyl)-2-(4-sulphophenyl)-2H-tetrazolium (MTS) and phenazine ethosulfate were added. The plates were incubated in a humidified atmosphere at 37°C in 5% CO_2_ for 2 h, and the bioreduction of MTS dye was evaluated by measuring the absorbance of each well at 490 nm using a microplate absorbance reader (iMark Microplate Reader; Bio- Rad Laboratories, Inc., Hercules, CA, USA). Cell growth inhibition was expressed as a percentage (mean ± SE) of the absorbance of the control cells.

### Anti- and pro-oxidant activity

To evaluate antioxidant activity, DPPH assay was used. The antiradical efficiency of the sample was evaluated by the DPPH stable radical method [11, 12]. 100 μl of sample, the essential oil of *C*. *juttae* at different concentrations, was added to aliquots (3.9 mL) of a solution made up with DPPH (4.8 mg) in MeOH (200 ml), and the mixture was incubated for 1 h at room temperature in the dark. Then the absorbance was measured at 517 nm using a UV-VIS spectrophotometer. The initial concentration of DPPH was approximately 60 μM. Lower absorbance values of reaction mixture indicate higher free radical scavenging activity. The results were plotted as the percentage of absorbance disappearance at 517 nm [(1-A/A_0_) × 100] against the amount of sample divided by the initial concentration of DPPH. Each point was acquired in triplicate. ED_50_ corresponds to micrograms of fraction able to consume half the amount of free radical divided by micromoles of initial DPPH. The results were expressed as antiradical capacity (ARC), which is the inverse of ED_50_.

Trolox (6-hydroxy-2,5,7,8-tetramethyl-chroman-2-carboxylic acid) curve was used as the positive control. Pro-oxidant activity was examined by cell counting, adding N-acetyl-L-cysteine (NAC), an antioxidant molecule, 1 h before essential oil. Data were expressed as mean ± standard error (SE) of at least three different experiments performed in duplicate. All the chemicals were supplied by Sigma Aldrich srl, Milan, Italy.

### Extraction of cellular RNA and reverse transcription-quantitative PCR (RT-qPCR)

Total RNA was extracted from cell lines using TRIzol reagent (Invitrogen Life Technologies). For the evaluation of gene expression, RNA was reverse transcribed using a high capacity complementary DNA (cDNA) reverse transcription kit (Applied Biosystems Life Technologies). The resulting cDNAs were subjected to real-time RT-PCR using the TaqMan Gene Expression Master Mix kit (Applied Biosystems Life Technologies) in triplicates. The PCR cycling conditions were as follows: Denaturation at 50°C for 2 min, annealing at 95°C for 10 min, followed by 40 cycles of 95°C for 15 sec and extension at 60°C for 60 min. The running of the samples and data collection were performed on a StepOne AB Real Time PCR system (Applied Biosystems Life Technologies). β-actin was used as an internal standard. The specific primers used were as follows: Survivin Hs00153353, XIAP Hs00236913, Bcl-2 Hs00236329, ABCB1 Hs00184005 (Applied Biosystems Life Technologies).

Relative expression was calculated using the comparative Ct method [ΔCt = Ct_(target gene)_-Ct_(housekeeping gene)_]. Where Ct was the fractional cycle number at which the fluorescence of each sample passed the fixed threshold. Fluorescence was measured at 515–518 nm using StepOne AB Real Time PCR System software (Applied Biosystems Life Technologies). The ΔΔCt method was used to determine gene expression levels. ΔΔCt was calculated using the formula: ΔΔCt = ΔCt_(each sample)_-ΔCt_(reference sample)_. Fold change was calculated using the 2^-ΔΔCt^ equation.

### Western blotting

Whole-cell lysates were obtained from breast cancer cells using RIPA buffer (Santa Cruz Biotechnology Inc., Dallas, TX, USA) and 25 μg protein was subjected to 10% SDS-PAGE and transferred to Hybond-P membranes (GE Healthcare Europe GmbH, Freiburg, Germany). Filters were incubated with primary antibodies raised against GAPDH (0411) (sc-47724 mouse anti-human polyclonal antibody 1:10000), Bcl-2 (sc-509 mouse anti-human monoclonal antibody 1:200) from Santa Cruz Biotechnology Inc., Dallas, TX, USA, Inc, XIAP (#2042, rabbit anti-human antibody, 1:500) from Cell Signaling Technology, Danvers, MA, Survivin (NB500-201, rabbit anti-human polyclonal antibody, 1:2000) from Novus Biologicals, Littleton, CO. Immunoblots were quantified by densitometry and results were expressed as arbitrary units (protein/GADPH).

### NF-κB activation

The DNA-binding capacity of NF-κB (p65 subunit) was determined in the nuclear extracts of MDA-MB-231 and SUM 149 cells using the TransAM NF-κB and Nuclear Extract kits (Active Motif, Carlsbad, CA, USA) according to the manufacturer's instructions. The cells were treated with 46 μg/ml and 64 μg/ml, respectively, of the essential oil for 24 h. Briefly, the determination of binding capacity was based on a 96-well plate, upon which an oligonucleotide containing the NF-κB consensus binding site (5'-GGGACTTTCC-3') was immobilized. Activated NF-κB contained in the extracts is able to specifically bind to this nucleotide. NF-κB bound to the oligonucleotide may subsequently be detected using an antibody directed against an epitope on p65 (polyclonal rabbit anti-human; cat. no. 40096; 1:1000; Active Motif), accessible only when NF-κB is bound to its target DNA.

Subsequently, the addition of a horseradish peroxidase-conjugated secondary antibody provided a sensitive colorimetric readout that may be quantified by densitometry (iMark Microplate Reader; Bio-Rad Laboratories, Inc.). The specificity of the assay was confirmed by simultaneous incubations in the presence of excess, non-immobilized consensus oligonucleotides, as a competitor, or of a mutated consensus oligonucleotide. The results were expressed as arbitrary units: one unit indicated the DNA binding capacity exerted by 2.5 μg whole cell extract from Jurkat cells (positive control for NF-κB p65 activation; Active Motif) (stimulated with 12-O-tetradecanoylphorbol-13-acetate and calcium ionophore) per microgram of protein from the nuclear extracts.

### Statistical analysis

Results of bioassays are given as means ± standard error (SE). Statistical analysis was carried out according to Poma *et al*. [13] by analysis of variance (one-way ANOVA) followed by Tukey’s test. Statistica ver. 12 (StatSoft Inc. 1984–2014) was used as software for the analyses.

## Results

The yield in essential was 0.002%. The plants from which the leaves were collected completed flowering and produced viable seeds. In the year 2018 the plants produced new leaves and the growth was not affected by the previous year removal of leaves.

### Chemical composition

In the essential oil of *C*. *juttae*, considering compounds with mass spectral similarities ≥ 70% in respect to our libraries, we detected 39 compounds (80% of the whole composition). Of these, 3 were classified as unknowns while 36 belonged to 18 different class and functional group of compounds.

**Fig 1:**
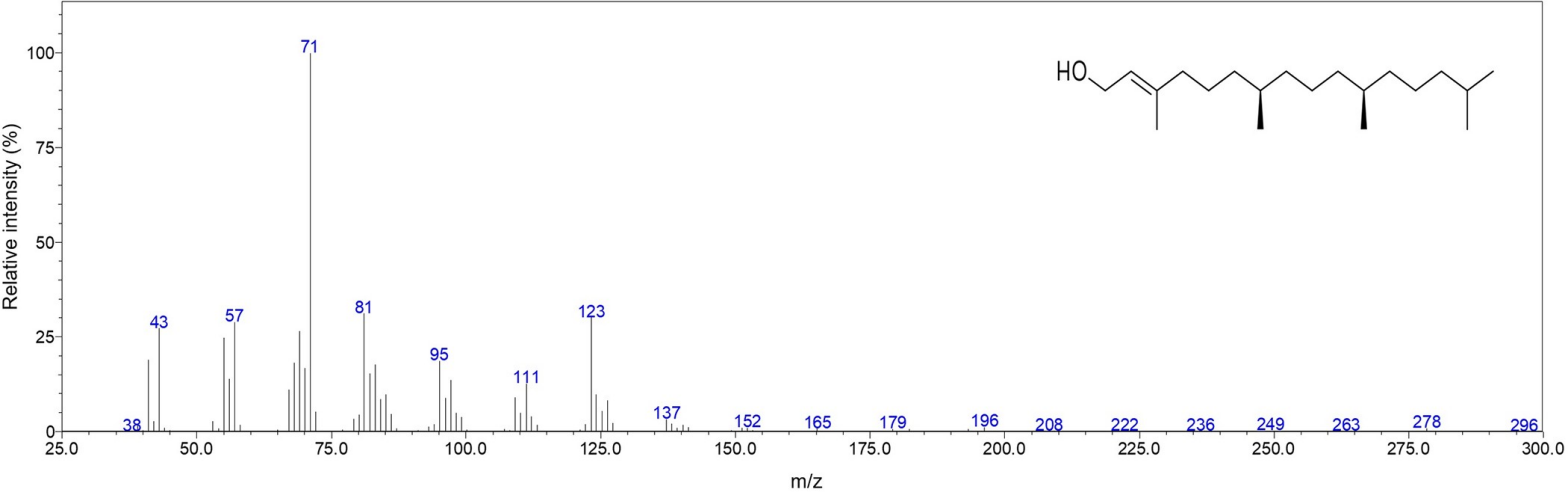
Mass spectra and chemical structure of phytol found in the present study.

Overall, the composition was dominated by Terpenenoid compounds with 64.9%, followed by Aliphatic Acids (7%) and Aliphatic Alkanes (2.9%). The most abundant compounds (> 4.0%) were phytol (29.6%; Fig 1), neophytadiene (6.6%) with its isomer III (4.6%), hexadecanoic acid (5.5%), 3-(2,6,6-Trimethyl-1-cyclohexen-1-yl)-2-propenal (5.5%) and isophytol (4.6%), contributing together 56.4% of the total composition accounting to 80.3%.

Nine compounds were detected in relative amounts between 1 and 4% (heptacosane, tetradecanoic acid, 1-(2,3,6-trimethylphenyl)-2-butanone, piperitenone, (*E*)-β-damascenone and carvacrol), whereas 24 compounds were found in amounts < 1% (Table 1).

**Table 1:** Essential oil composition of *C*. *juttae*. Compounds belonging to the same chemical class and functional group are arranged according to Kovats Retention Indices (KRI) of the ZB-5 column.

KRI	Compound	Relative amount (%)	MS Similarity (%)
	***Aliphatic Alcohols***		
898	2-Heptanol	0.2	96
1096	1-Octanol	0.1	93
	***Aliphatic Aldehydes***		
858	(*E*)-2-Hexenal	0.7	96
1104	Nonanal	0.6	97
	***Aliphatic Alkanes***		
2300	Tricosane	0.1	90
2500	Pentacosane	0.3	85
2700	Heptacosane	2.5	90
	***Aliphatic Esters***		
1190	Butyl hexanoate	0.1	91
1386	Hexyl hexanoate	0.4	92
2105	Methyl linolenate	0.5	95
	***Aliphatic Acids***		
1758	Tetradecanoic acid	1.5	94
1961	Hexadecanoic acid	5.5	92
	***Aromatic Esters***		
1780	Benzyl benzoate	0.1	94
	***Aromatic Hydrocarbons***		
1029	1,4-Diethylbenzene	0.1	90
1161	1,2,4,5-Tetramethylbenzene	0.1	88
	***Aromatic Ketones***		
1593	1-(2,3,6-Trimethylphenyl)-2-butanone	1.1	82
	***Diterpene Alcohols***		
1950	Isophytol	4.6	96
2118	Phytol	29.6	97
	***Monoterpene Alcohols***		
1100	Linalool	0.1	95
1303	Carvacrol	4.0	80
	***Monoterpene Ethers***		
1077	(*Z*)-Linalool oxide furanoid	0.1	94
1093	(*E*)-Linalool oxide furanoid	0.1	92
	***Monoterpene Hydrocarbons***		
981	*p*-Cymene	0.01	80
1034	Limonene	0.1	96
	***Monoterpene Ketones***		
1249	Pulegone	0.7	96
1353	Piperitenone	1.7	96
	***Irregular terpene Aldehydes***		
1410	3-(2,6,6-Trimethyl-1-cyclohexen-1-yl)-2-propenal	5.5	81
	***Irregular terpene Ketones***		
1394	(*E*)-*β*-Damascenone	1.6	94
1456	Geranylacetone	0.3	89
	***Sesquiterpene Alcohols***		
1600	Fokienol	0.3	90
1673	*α*-Cadinol	0.6	89
	***Sesquiterpene Ethers***		
1605	Caryophyllene oxide	0.7	94
	***Sesquiterpene Hydrocarbons***		
1693	Cadalene	0.2	87
1840	Neophytadiene	6.6	94
1865	Neophytadiene, Isomer II	2.5	94
1883	Neophytadiene, Isomer III	4.6	96
	***Unknowns***		
1206	*m/z*: 131(100), 159(72), 57(61), 91(58), 41(56), 55(41)	0.4	71
1310	*m/z*: 105(100), 120(79), 91(65), 119(58), 92(32), 77(27)	1.1	79
2142	*m/z*: 79(100), 253(62), 67(61), 93(50), 80(44), 41(42)	1.1	79
	**Total**	**80.3**	

### In vitro anticancer and pro-oxidant activity

**Fig 2:**
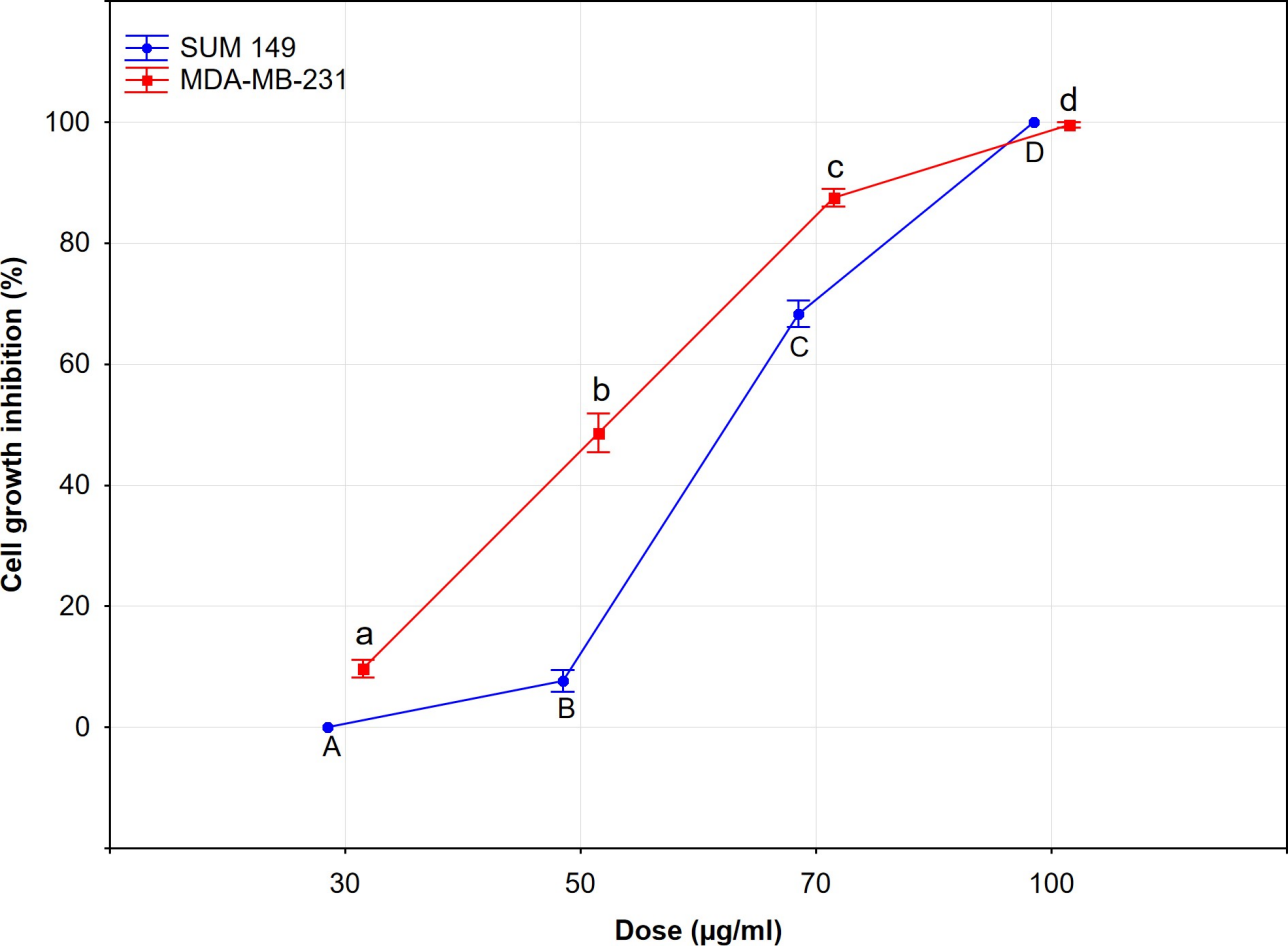
Cytotoxic activity of *C*. *juttae* essential oil on cancer cell lines. Cell viability was assessed by MTS. Data are expressed as mean ± standard error (SE) of at least three different experiments performed in triplicate. Different letters represent significant differences in cytotoxic activity among the concentrations of each cell line (Tukey test, p < 0.05).

We examined the cytotoxic activity of *C*. *juttae* essential oil on MDA-MB-231 and SUM 149 cells by 3-(4,5-dimethylthiazol-2-yl)-5-(3-carboxymethoxyphenyl)-2-(4-sulphophenyl)-2H-tetrazolium (MTS) assay. The essential oil of *C*. *juttae* reduced cell growth in a dose dependent way, in particular the cell line more sensitive was MDA-MB-231 (Fig 2). The essential oil did not show cytotoxic activity on the non-tumorigenic cell line 1-7HB2. The cell growth inhibition was: 0% at 30 μg/ml; 10 ± 2% at 50 μg/ml; 17.5 ± 1.5% at 70 μg/ml; and 23 ± 2% at 100 μg/ml.

Since many natural compounds are provided with anti- or pro-oxidant activity, we analyzed if the antitumor activity of the *C*. *juttae* oil was due to pro-oxidant activity. The cells were treated with the antioxidant N-acetyl-L-cysteine (NAC) at 2mM 1 h before exposure to the essential oil at the corresponding IC_50_ values. In both cell lines the addition of NAC reduced cytotoxic activity, in particular the reduction was more evident in MDA-MB-231 cell line (Table 2).

**Table 2:** Results of cell counting analysis in the two cell lines following treatment with antioxidant N-acetyl-L-cysteine (NAC) at 2mM before exposure to the essential oil at the corresponding IC_50_. Data are expressed as mean ± standard error (SE).

Cell lines and treatments	Cell viability (%)
***MDA-MB-231***	
+ NAC 2 mM^a,^**	73.6 ± 2.6
+ essential oil of *C*. *juttae* 46 μg/mL^b,^**	45.0 ± 4.0
+ NAC 2 mM + essential oil of *C*. *juttae*^a,^**	72.6 ± 0.4
***SUM 149***	
+ NAC 2 mM^a^	98.6 ± 1.4
+ essential oil of *C*. *juttae* 64 μg/mL^b,^**	52.0 ± 6.0
+ NAC 2 mM + essential oil of *C*. *juttae*^ab,^*	74.4 ± 4.4

Different letters (a and b) in the column of the cell lines and treatments represent significant differences among the treatments of each cell line

differences when treatments are compared to the control

*p < 0.05

**p < 0.01.

In addition, the analysis carried out by DPPH (2,2-diphenyl-1-picrylhydrazyl) reduction assay indicated that the essential oil does not possess antioxidant activity, since it was not identified the efficient dose (ED_50_) of the essential oil of *C*. *juttae* (Table 3). These results indicate that the mechanism involves an at least partially pro-oxidant effect.

**Table 3:** Results of antioxidant activity performed with DPPH method (DPPH free radical scavenging activity).

Compound	ED_50_	ACR (1/ED_50_)
Trolox	68 μg/ml	0.015
Essential oil	>100μg/ml	-

### Inhibition of NF-κB activation

**Fig 3:**
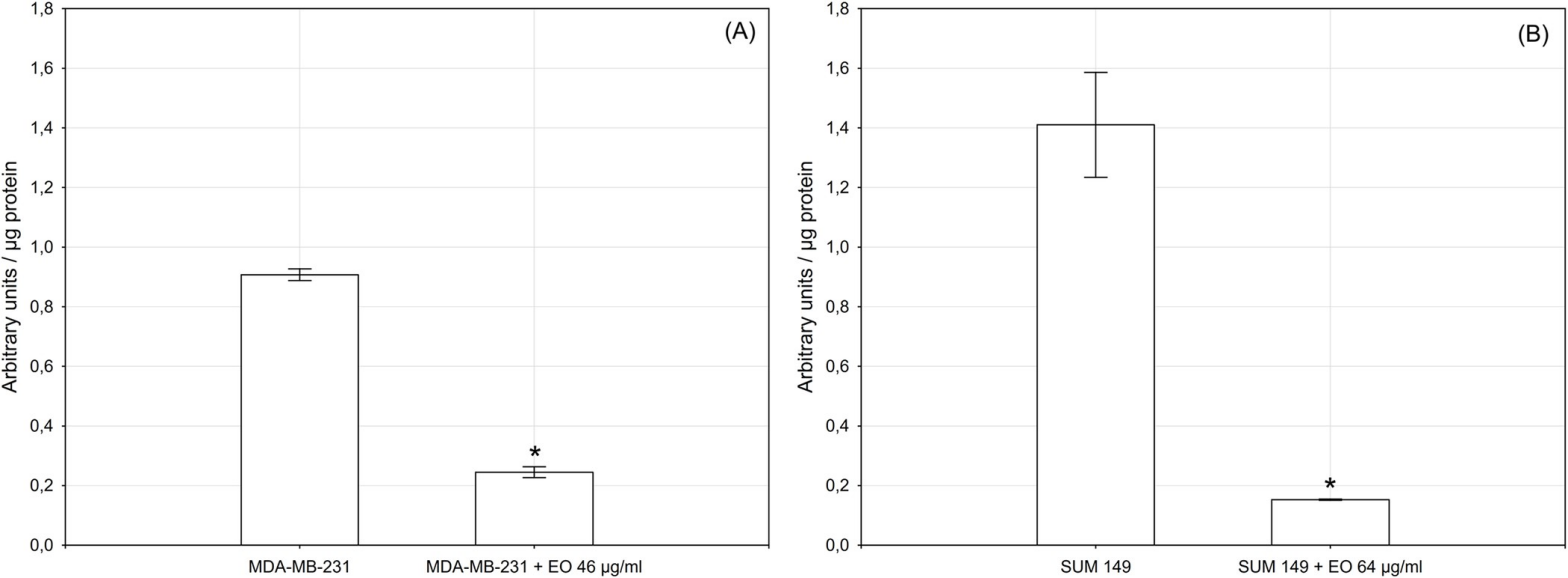
**NF‑κB (p65 subunit) DNA binding capacity in nuclear extracts of MDA-MB-231 cells (A) and of SUM 149 cells (B).** The cells were treated for 24 h with *C*. *juttae* essential oil (EO) (46 and 64 μg/ml, respectively). Results (mean ± standard error of two experiments carried out in duplicate) are expressed as arbitrary units/μg protein of cells nuclear extracts. Statistical differences are *p < 0.05 vs. control.

In order to investigate the molecular mechanism underlying the antitumor activity of the essential oil, we analyzed the effects of the essential oil to NF-κB activation by TransAM assay. MDA-MB-231 and SUM 149 cells were treated with the essential oil at the corresponding IC_50_ values for 24 h: in both cell lines we observed a strong reduction of NF-κB DNA-binding activity (Fig 3).

**Fig 4:**
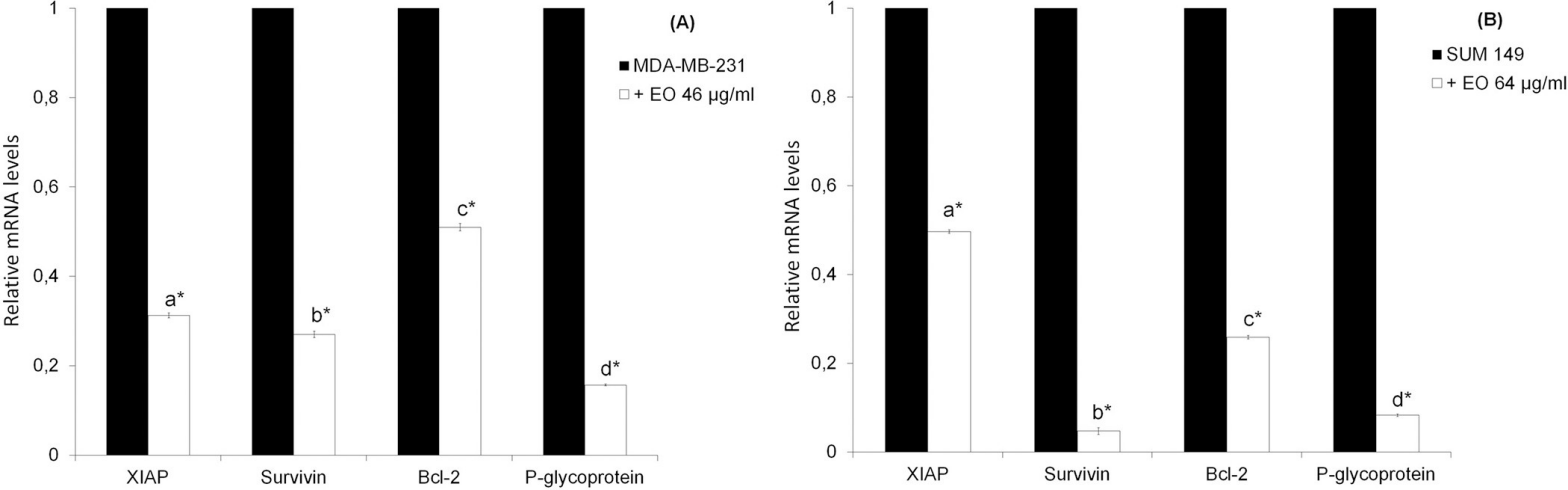
**Genes mRNA expression levels by quantitative polymerase chain reaction in MDA-MB-231 cells (A) and SUM 149 cells (B).** The cells were treated for 24 h with *C*. *juttae* essential oil (EO) (46 and 64 μg/ml, respectively). Data are expressed as mean ± standard error (SE) of two different experiments. Different letters (a, b, c and d) in the column of the cell lines and treatments represent significant differences (p < 0.05) among the treatments of each cell line; *differences when treatments are compared to the control (p < 0.05).

In view of this, the treatment with the essential oil also caused a substantial mRNA decrease of some NF-κB target genes, in particular antiapoptotic factors such as Survivin, XIAP, Bcl-2 and the multidrug efflux transporter P-glycoprotein, encoding by ABCB1 gene (Fig 4).

**Fig 5:**
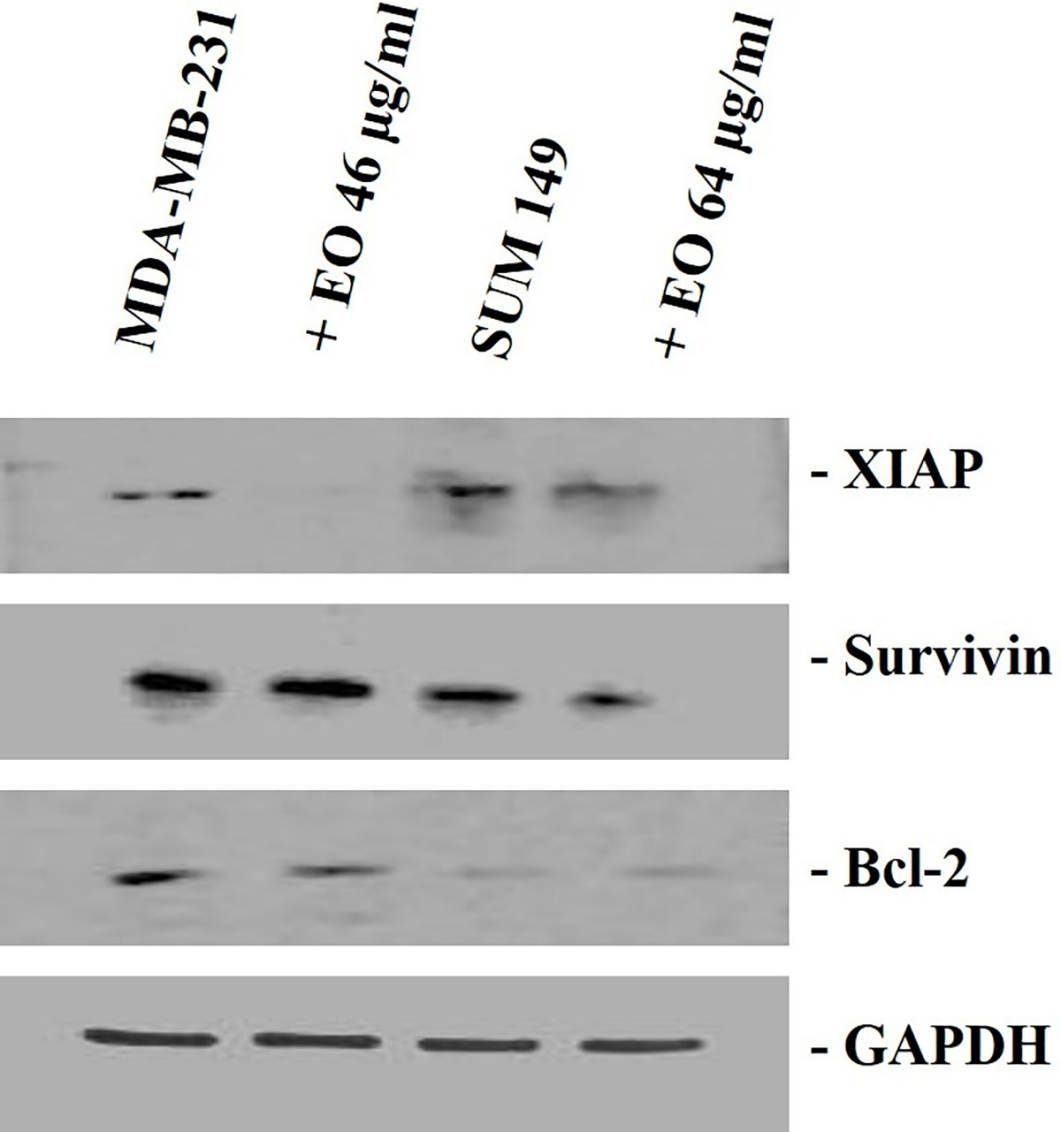
Western blotting analysis in MDA-MB-231 cells and SUM 149 cells. The cells were treated for 24 h with *C*. *juttae* essential oil (EO) (46 and 64 μg/ml, respectively). The data shown are the results of a representative experiment.

In both cell lines we found a reduction also at protein level of anti-apoptotic factors (Survivin, XIAP and Bcl-2) under essential oil treatment (Fig 5).

The strong reduction of mRNA level of P-gp under treatment with essential oil in our cell lines is significant but the analysis of the drug resistance implies further experimental evaluations that are in progress. However, it is known that the expression of P-gp is also regulated by post-translational events, such as post-transcriptional glycosylation and membrane localization of P-gp [14].

## Discussion

Essential oils and their chemical constituents have potential roles and uses to prevent and treat human disease. The role of these natural products has been discussed with regard to the prevention and treatment of cancer, cardiovascular diseases, and as antibacterial, antiviral, and antioxidant agents, among others [15].

Since *C*. *juttae* is a plant species adapted to dry environments with high temperature, high light intensity, and low nutrient and water resources the risk of damage by abiotic and biotic factors can be easy to occur. However, by the biosynthesis of a wide range of secondary metabolites this species, growing in nature in prohibitive habitats, can develop defensive strategies against several biotic and abiotic factors.

Our chemical composition indicates that *C*. *juttae* essential oil constituents potentially may play several biological roles. Among others, terpenoids contributing to more than 60% of *C*. *juttae* composition are the largest group of plant chemicals which have multiple ecological roles, such as: (1) defense against generalist and specialist herbivores, (2) defense against fungi and bacteria, (3) attraction of pollinators and seed dispersers, (4) allelopathic agents that inhibit seed germination, and (5) protection against abiotic factors (e.g. with reactive troposphere gases) [16, 17]. According to Gershenzon and Dudareva [18] considering the large number of terpenoids produced in nature, very little is known about their mode of action at the molecular level although their highly lipophilic nature suggests that they act on the cell membranes and that their toxicity is caused by loss of chemiosmotic control.

Many plant-derived active principles have gained attention as efficient anticancer agents against TNBCs, with fewer adverse side effects. In this work for the first time we described the strongly inhibitory effect of *C*. *juttae* essential oil on NF-κB activation in two TNBC cell lines characterized by a strong expression of the transcriptional factor. Consequently, the inhibition of transcriptional activity due to essential oil leads to a significant decrease of some transcriptional target genes such as Survivin, XIAP, Bcl-2 and P-glycoprotein [19, 20, 21, 22].

Moreover, in the same cancer models, the essential oil showed antitumor capacities in the terms of cytotoxic and pro-oxidant activities and partially pro-apoptotic effects proven by preliminary cytofluorimetric analysis by propidium iodide where only a modest block in preG0-G1 position dose-dependent is observed in all cell lines and also shown in other studies [23]. It is very interesting to note that the essential oil exerts an inhibitory effect on the transcription of the P-glycoprotein the ABCB1 multi-drug efflux pump (MDR). P-glycoprotein is overexpressed in TNBCs and analysis of the MDR reversal activities of essential oil request further analyses [24, 25, 26]. In sight of this plant-derived compounds in combination with classical chemotherapeutic agents could be more efficient in the treatment of TNBCs, possibly with lesser side effects.

Our results on the chemical composition of the *C*. *juttae* essential oil shown that Phytol is the main compound (29.6%). Based on these data, it could be hypothesized that the biological activities of essential oil in our TNBC cells, are accountable mainly to phytol which in combination with other terpenoids can act with synergic effects. On the other hand, the antitumor activity of this compound has been already described by Islam *et al*. [27] in different type of cancer models, such as human gastric adenocarcinoma, lymphoid leukemia, hepatocellular carcinoma and also in one triple negative breast cancer cell line. There are other evidences that showed how the immunomodulatory and antitumor effects of the Phytol can be attributed precisely to the action on NF-κB [27].

## Conclusions

Our studies have shown that essential oil of *C*. *juttae* exhibits the remarkable property to disturb NF-κB molecular pathway and it could be useful to sensitize the TNBCs to the conventional antitumor drugs.
